# Effect of Docosahexaenoic Acid on Apoptosis and Proliferation in the Placenta: Preliminary Report

**DOI:** 10.1155/2015/482875

**Published:** 2015-08-03

**Authors:** Ewa Wietrak, Krzysztof Kamiński, Bożena Leszczyńska-Gorzelak, Jan Oleszczuk

**Affiliations:** Department of Obstetrics and Gynecology, Medical University of Lublin, Jaczewskiego 8 Street, 20-954 Lublin, Poland

## Abstract

*Introduction.* Observational studies confirm a higher incidence of preeclampsia in patients with low erythrocyte concentrations of omega-3 fatty acids. Observations point to an association of disorders of pregnancy, such as intrauterine growth restriction (IUGR) and preeclampsia, with excessive apoptosis. One potential mechanism of action of docosahexaenoic acid (DHA) promoting a reduction in the risk of pathological pregnancy may be by influencing these processes in the placenta. *Materials and Methods.* We investigated 28 pregnant women supplemented with a fish oil product containing 300 mg DHA starting from pregnancy week 20 until delivery (DHA group). The control group consisted of 50 women who did not receive such supplementation (control group). We determined the expression of Ki-67 and p21 as markers of proliferation and caspase 3 activity as a marker of apoptosis and DHA levels in umbilical cord blood. *Results.* Caspase 3 activity was significantly lower in the DHA group in comparison to the control group. Umbilical cord blood DHA concentration was higher in the DHA group. The expression of the proteins p21 and Ki-67 did not differ significantly between the groups. *Conclusions.* We observed an association between DHA supplementation and inhibition of placental apoptosis. We did not find an association between DHA and proliferation process in the placenta.

## 1. Introduction

The use of omega-3 acids in pregnant women dates back to the 1980s, when Olsen conducted the first observational study of the effect of dietary fish oil on the pregnancy outcome [1]. Further research revealed that docosahexaenoic acid (DHA) was responsible for many of observed effects. DHA is a long-chain fatty acid belonging to the omega-3 group. These essential unsaturated fatty acids need to be supplied in the diet as they cannot be synthesised by the body. Fatty marine fish are the richest source of long-chain fatty acids. At present, many international scientific societies, including the Polish Gynaecological Society, recommend the use of DHA for the prevention of premature labour [2, 3]. They are based on meta-analyses and large RCT studies conducted by Makrides et al. and Carlson et al. [4, 5]. Currently in Australia a large clinical trial takes place among 5500 women. The aim of this study is to observe the effects of DHA from fish oil (800 mg) on the risk of preterm delivery and adverse pregnancy outcome [6]. The role of DHA in the development of the central nervous system and its influence on cognitive processes have also been appreciated by paediatricians, who recommend DHA consumption by pregnant women and breast-feeding mothers. The prenatal and early postnatal period are critical for the development of the infant brain [7]. An animal model has shown that DHA plays a role in the prevention of cerebral hypoxia. If this effect is confirmed in human studies, it will be a high clinical significance [8]. Studies investigating the link between DHA consumption and the development of preeclampsia or postpartum depression have not been consistent [5, 9]. Available data do not make it clear which doses should be used in the presence of specific disorders of pregnancy since various studies have used a wide range of daily dosages from 500 mg to 2.7 g [10]. In spite of so many unknowns, many experts agree that DHA should be regarded as an important component of a pregnant woman's diet and an element of nutritional programming [11]. New data from animal studies and a cell model indicate a protective effect of omega-3 acids, and particularly DHA, on central nervous system cells [12]. It has been proved that this effect is caused by antiapoptotic activity. In women, apoptosis plays a significant role in placentation. Intense apoptosis in the placenta is believed to be a primary factor in the pathogenesis of such disorders of pregnancy as preeclampsia and IUGR [13]. Increased apoptosis in peripheral blood is also associated with the destruction of fetal DNA fragments [14]. Intense placental apoptosis leads to cell destruction, decreasing the area available for oxygen exchange and transfer of nutrients and metabolites and, later, leading to hypoxia and, consequently, the development of disorders of pregnancy [15]. Animal studies of the effect of DHA on apoptosis and observations of excessive apoptosis in IUGR and preeclampsia in conjunction with observational evidence of a correlation between the incidence of these pathologies and low DHA levels prompted us to study the effect of DHA on placental apoptosis in pregnant women.

## 2. Objective

The present paper aims to improve our understanding of the effect of DHA on cellular proliferation (Ki-67 and p21 expression) and to assess placental apoptosis (caspase 3 activity) in women with normal uncomplicated pregnancies without disorders such as IUGR, preeclampsia, or premature delivery. The correlation between DHA supplementation and cord blood DHA concentration is also investigated.

## 3. Material and Methods

The study involved a group of 28 parturient women who were supplemented with fish oil from pregnancy week 20 until delivery. The women from the supplementation group gave birth at the Department of Obstetrics and Perinatology, Independent Public Teaching Hospital number 4 in Lublin, between March and August 2012. There was a control group of 50 women who did not receive supplementation. Women were enrolled in the study on the basis of their history of supplementation in pregnancy. The women in the supplementation group took one capsule of a supplement containing 300 mg DHA (Prenatal DHA, Holbex). The supplementation and control groups were matched for age, fertility, parity, and pregnancy age. All women in the study had single pregnancies with alive fetuses, were negative for clinical and biochemical markers of inflammation, did not smoke tobacco, and had no chronic medical conditions that could lead to angiopathy. The study protocol was approved by the Ethical Committee at the Medical University in Lublin.

DHA concentration was determined in all patients in the serum of venous cord blood. The profile of fatty acids in the spectrum range for DHA was analysed by gas chromatography using for lipids separation Folch method [16].

The p21 protein is a potent inhibitor of cyclin-dependent kinases. P21 protein binds to cyclin complexes to inhibit their activity, thus acting as a cell cycle regulator in phase G1. P21 expression was determined in freshly frozen placental tissue sections by immunohistochemistry using standard sets of monoclonal antibodies and an avidin-biotin detection system from Dako [17]. Positive p21 reaction was defined as the fluorescence of more than 10% of the cells at a magnification of 100x.

The expression of the Ki-67 protein was detected with the PP-67 standard murine anti-human monoclonal antibody kit (Sigma-Aldrich) [18]. The Ki-67 antigen is found in the cell nucleus, especially in the nucleoli during the interphase. It is the protein marker of cellular proliferation. It was determined immunohistochemically in freshly frozen sections based on detection system for biotinylated samples (Dako) [19]. Ki-67 positivity was defined as the presence of a positive immunohistochemical reaction in more than 30% of cells per field at a magnification of 100x.

Caspase 3 activity was determined by colorimetry in homogenates of slices of full sections through placentae obtained after delivery. The commercially available caspase 3 colorimetric assay kit code CASP-3-C (Sigma-Aldrich, USA) was used. Caspase 3 activity was converted to *μ*mols of p-nitroaniline release per minute (min) per millilitre (mL) of a placental cell lysate or a positive control [20].

Differences in the different assay results between the groups were analysed with the Mann-Whitney *U* test (*P*(*U*)). The correlation between cord blood DHA levels and the apoptosis index was analysed by estimating Spearman's rank correlation coefficients. Differences were considered statistically significant at *P* < 0.05.

## 4. Results

**Figure 1:**
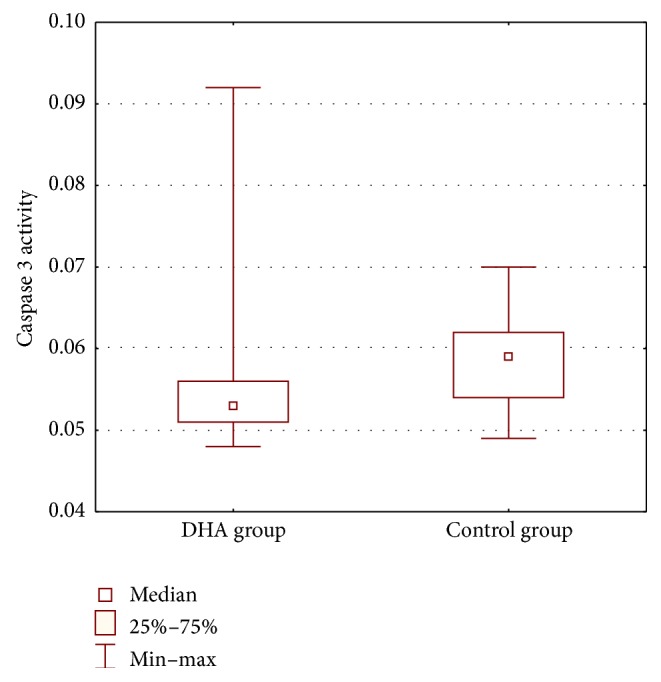
Average activity of caspase 3 (*μ*mol pNA/min/mL) in both analyzed groups (*P*(*U*) = 0.0001).

The statistical analysis of the data obtained in the supplementation versus control groups did not reveal statistically significant differences in the distribution of results with regard to age, duration of pregnancy, and body weight at birth between the groups. The supplementation and control groups were assumed to be comparable with regard to these indices. There were significant differences between the groups with regard to placental weight (*P*(*U*) = 0.0071), which was significantly higher in the control group. Apgar scores did not differ significantly between the groups (*P*(*U*) = 1.0000). The index of apoptosis, that is, caspase 3 activity, was significantly different between the groups (*P*(*U*) = 0.0001) (Figure 1): it was significantly lower in the DHA supplementation group as compared to the control group. The clinical details of the patients and their children and caspase 3 activity are presented in Table 1.

**Figure 2:**
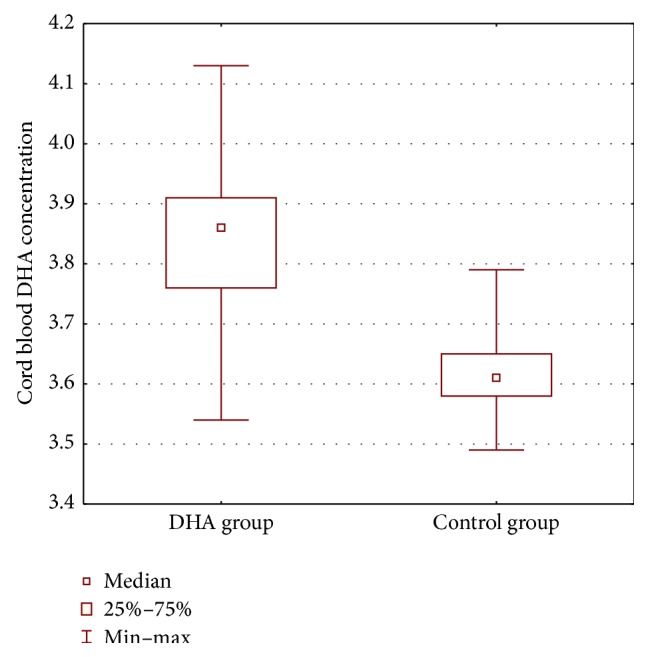
Average concentrations of DHA (ng/*μ*L) in the serum of venous cord blood in both analyzed groups (*P*(*U*) = 0.0001).

**Figure 3:**
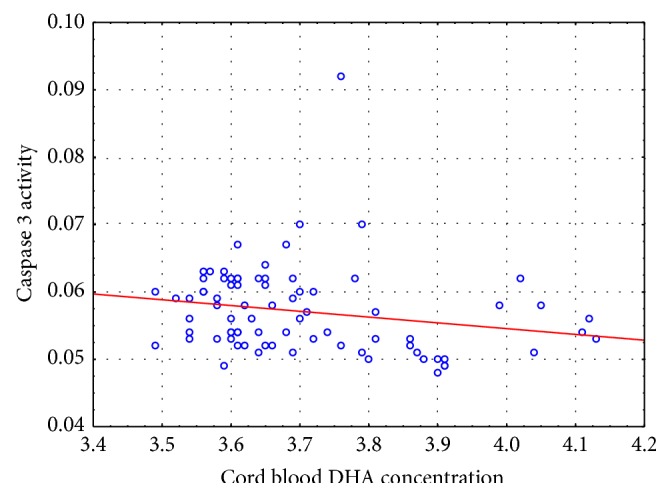
Distribution of caspase 3 activity in the placenta depending on the concentration of DHA in cord blood.

DHA concentration in the serum of venous cord blood differed significantly between the groups (*P*(*U*) < 0.0001). Cord blood DHA levels were higher in the DHA supplementation group (Figure 2). Overall, there was a highly significant negative rank correlation between cord blood DHA levels and the index of apoptosis (*P* < 0.01). Overall for all values we have Spearman's correlation −0,3082. For DHA group we received −0,0670 and for control group 0,0622, respectively. This finding means that as DHA levels increase in cord blood caspase 3 activity decreases (Figure 3). The correlation was calculated with Spearman's coefficient, which is robust for nonnormal distributions and outliers.

Expression of the proteins p21 and Ki-67 was not significantly different between the groups (*P* > 0.05). For p21 protein we observed a positive reaction in 17% and 15% of cells for the DHA and control group, respectively. Positive reaction for Ki-67 was received for 21% and 20% cases for DHA and control group, respectively. It indicates that the DHA dose used in the study had no effect on placental proliferation in the patients.

## 5. Discussion

The diet of citizens of the Western countries is characterised by a predominance of omega-6 acids over omega-3 acids, which may lead to intensified proinflammatory activity, narrowing of vessels, a prothrombotic effect, and, consequently, preeclampsia in pregnant women. Observational studies confirm greater incidence of this pathology in women on a fish-deficient diet or in those with low omega-3 acid levels in erythrocytes [21, 22]. In a 2011 meta-analysis, Duley pointed to the lack of sufficient data to assess the effectiveness of fish oil in the prevention and treatment of preeclampsia [9]. In contrast to this, the Canadian Hypertension Society has published recommendations on nonpharmacological management and prevention of hypertension, advising the use of fish oil in cases of hypertension in pregnant women. They particularly recommend fish oil supplementation in women from risk groups who experienced hypertensive episodes in early pregnancy [23]. The latest data indicate that women with preeclampsia have a reduced pool of long-chain fatty acids and reduced placental synthesis and fetal transport. That is why the supplementation of omega-3 fatty acids may improve their status both in the women and in children born to mothers with preeclampsia [24]. One potential mechanism of action of DHA promoting a reduction in the risk of pathological pregnancy may be by influencing cellular apoptosis and proliferation in the placenta.

Placental apoptosis is physiological in normal pregnancy and intensifies over time. Placental apoptosis has been particularly studied in two pathologies of pregnancy: preeclampsia and IUGR [13]. A finding shared by both of these conditions is intense placental apoptosis, which is probably a key mechanism leading to placental dysfunction. The attenuation of proliferation in pathological pregnancy is not an established fact, with published studies presenting contradictory results [25–27].

The effect of omega-3 acid supplementation on proliferation and apoptosis in various cells of the placenta has been described to date in only one paper by Klingler et al. [28]. The authors put forward the hypothesis that greater proliferation could be expected towards the end of pregnancy on the basis of earlier reports that high dosages of DHA can prolong pregnancy and increase infant birth weight [29]. Klingler et al. reported that supplementation did not influence the duration of pregnancy or the birth weight of the infants. There were also no changes in apoptosis between the groups. Only a group using fish oil with folate demonstrated an effect on proliferation. Our results do not confirm an effect of DHA on cell proliferation, which could be due to an insufficient dosage used (300 mg DHA compared to 500 mg in the study by Klingler et al.). At the same time, we did demonstrate an influence of DHA supplementation on apoptosis. The effect on proliferation observed in Klingler's study might have been due to the synergistic action of DHA and folate in the study preparation. Even though DHA had no effect on proliferation in our study, placental weight in the DHA group was significantly higher, which may be attributed to DHA-mediated reduction of oxidative stress as observed in an animal model by Jones et al. [30].

Importantly, DHA-mediated inhibition of neuronal apoptosis has been demonstrated in an animal model. In a Japanese study, pregnant rats, some of which had been fed a DHA-rich diet, were placed in hypoxia. The DHA group demonstrated fewer apoptotic neurons, which may be a sign of neuroprotection by inhibition of oxidative stress in nerve cells [8]. The latest reports also indicate an inhibitory effect on neuronal apoptosis following the addition of omega-3 acids to the diet in postnatal hyperoxia [12].

Our report also describes an effect of daily supplementation of 300 mg DHA on the levels of this compound in cord blood, which were significantly higher than in the control group. In previous studies, higher DHA cord blood levels were obtained at higher DHA dosages [31, 32]. According to earlier data, a dosage of 200 mg did not raise DHA levels in cord blood [33]. Cord blood DHA levels are a marker not only of dietary DHA (including DHA supplementation) but also of DHA synthesis from its precursors, which is increased in pregnancy. Accordingly, the determination of DHA in cord blood appears to be the most appropriate measure of the amount of DHA reaching the fetus. Recent data suggest that cord blood DHA levels may be influenced by other factors than maternal DHA consumption, which may account for the different conclusions from the assessment of the infants in studies where DHA supplementation was the responsibility of the pregnant women [34].

We currently know little about the effect of DHA on placental proliferation and apoptosis, with only one paper of relevance to this issue in the global literature. A confirmation of our results by a study with a larger sample may in the future lead to the development of recommendations and prophylactic use of DHA to prevent primary disorders of pregnancy associated with too shallow placentation, that is, preeclampsia and IUGR.

Our findings are the outcome of a pilot study of a group of pregnant women with no history of disorders related to placental dysfunction. The limitations of our study comprise the dosage of 300 mg DHA, which was consistent with clinical recommendations at the time of the study. Current recommendations suggest the need to use higher doses of DHA at least 600 mg. New clinical trials at dose 800 mg are currently being designed. It seems that in our study too low dose was used, to assess the effect on apoptosis and proliferation rates. The second limitation was the small number of participants in this pilot study.

Accordingly, a continuation of this study should involve women at risk for these disorders and be conducted on a larger sample with higher DHA dosages. Since placentation is already influenced by DHA in the first trimester of pregnancy, it is advisable to conduct a study of DHA supplementation from the beginning of pregnancy [35]. Regarding the timing of supplementation we postulate that the effect of DHA, which improves the clinical outcome, is to prevent defective deep placentation. Therefore, the more early in pregnancy the DHA starts, the greater the effect on improving placentation is, making it possible to achieve a significant improvement in clinical outcomes. A limitation of our study was the lack of assessment of the effects of DHA supplementation on placentogenesis using placental bed biopsies, which so far has not been studied [35].

## 6. Conclusions

Cord blood DHA levels were significantly higher among the women who received DHA supplementation in comparison with the controls. We did not find an association between DHA and proliferation process in the placenta. The finding of reduced caspase 3 activity furnishes evidence for confirming the association of DHA supplementation with inhibition of placental apoptosis. The intensity of apoptosis was correlated with DHA levels in venous cord blood, which suggests an influence of this compound on the incidence of disorders of pregnancy associated with excessive apoptosis in cases of placental failure. Further research is necessary to more precisely determine the role of DHA in placenta-related disorders of pregnancy.

## Figures and Tables

**Table 1:** Clinical data and the activity of caspase 3 and the concentration of DHA in the serum of venous cord blood in patients from both analyzed groups.

Variable	Median	Min–max	95% CI	*P*(*U*) value
Maternal age (y)				
DHA group (*n* = 28)	25,5	19–39	25,2–26,7	**0,5399**
Control group (*n* = 50)	26	18–35	24,2–26,8	
Gestational age (w)				
DHA group (*n* = 28)	39,5	37,8–41,2	39,26–39,64	**0,2151**
Control group (*n* = 50)	39,20	37,6–41		
Birth weight (g)				
DHA group (*n* = 28)	3675	2990–4350	3589–3760	**0,8815**
Control group (*n* = 50)	3785	3170–4400	3725–3844	
Placenta weight (g)				
DHA group (*n* = 28)	510	440–650	497–522	**0,0071**
Control group (*n* = 50)	530	410–610	522–537	
Apgar score at 5 minutes (p)				
DHA group (*n* = 28)	10	8–10	9,87–10,13	**1,0000**
Control group (*n* = 50)	10	7–10	9,88–10,12	
Caspase 3 activity (*μ*mol pNa/min/mL)				
DHA group (*n* = 28)	0,053	0,0084–0,048	0,0511–0,0549	**0,0001**
Control group (*n* = 50)	0,059	0,0047–0,049	0,0582–0,0598	
Cord DHA (ng/*μ*L)				
DHA group (*n* = 28)	3,86	3,54–4,13	3,83–3,89	**0,0001**
Control group (*n* = 50)	3,61	3,49–3,79	3,60–3,62	
